# Design of and outcomes in a student-run free mental health clinic serving the uninsured in East Harlem

**DOI:** 10.1186/s12888-022-04112-w

**Published:** 2022-07-26

**Authors:** Samuel K. Powell, Alexandra Saali, Justin Frere, Elizabeth Magill, Hannah Krystal, Randal A. Serafini, Syeda Sultana, Brandon Dale, Muhammad Ali, Vedika Kumar, Debjyoti Datta, Josimar Hernandez-Antonio, Anne Aronson, Yasmin S. Meah, Vicki Gluhoski, Craig L. Katz

**Affiliations:** Icahn School of Medicine at Mount Sinai, New York, NY 10029 USA

**Keywords:** HEDIS, Psychiatry, Student-run free clinic, Immigrants, Patient outcomes

## Abstract

**Background:**

Safety-net clinics are an important source of low-cost or free mental healthcare to those with limited financial resources. Such clinics are often staffed by trainees in early stages of their career. Only limited data exist on best practices in treatment-implementation and on clinical outcomes attained in such clinics. The primary purpose of this article is to describe the design of an outpatient psychiatry student-run free clinic (SRFC) serving uninsured individuals in New York City’s East Harlem neighborhood and to analyze the quality of services provided and the clinical outcomes attained.

**Methods:**

The authors conducted a retrospective chart review of *n* = 69 patients treated in the EHHOP Mental Health Clinic (E-MHC) to describe the demographic and clinical characteristics of the study population. Utilizing Health Effectiveness Data and Information Set metrics, they estimated the likelihoods of patients meeting metric quality criteria compared to those in other New York State (NYS) insurance groups. The authors derived linear mixed effect and logistic regression models to ascertain factors associated with clinical outcomes. Finally, the authors collected patient feedback on the clinical services received using a customized survey.

**Results:**

Almost all patients were of Hispanic ethnicity, and about half of patients had more than one psychiatric disorder. The clinical service performance of the E-MHC was non-inferior on most measures examined. Factors associated with symptom improvement were the number of treatment sessions and certain demographic and clinical variables. Patients provided highly positive feedback on the mental healthcare services they received.

**Conclusions:**

SRFCs can provide quality care to vulnerable patients that leads to clinically meaningful reductions in psychiatric symptoms and is well-received by patients.

**Supplementary Information:**

The online version contains supplementary material available at 10.1186/s12888-022-04112-w.

## Background

A substantial proportion of those with psychiatric conditions do not receive adequate clinical care. A 2015 nationally representative survey found that 62% of adults with mental illness did not receive treatment and that health insurance status was a leading predictor of mental healthcare utilization [1]. The most recent data indicate that just over 11% of U.S. adults with mental illness remain uninsured and that this is a key contributor to the estimated 25% who reported that they were unable to receive the treatment they needed [2]. This challenge is compounded by the increased rates of under-insurance among those with psychiatric conditions compared to those without [3]. The proportion of individuals who are uninsured is particularly high among immigrants who have not met legal residency requirements [4]. In New York City alone, at least 40% of the estimated 2.2 million immigrants lack health insurance [5]. As a result, utilization of mental healthcare is particularly low among these individuals [6–8].

Safety-net clinics affiliated with academic medical centers are an important source of free or low-cost care to those with limited financial resources. These clinics are often staffed by unpaid volunteers, many of whom are students and trainees. There are currently over 200 student-run free clinics (SRFCs) which, in total, provided 115,000 patient visits in 2014 [9]. Not yet able to establish their own independent practices, students gain hands-on, supervised clinical experience by providing care to individuals unable to afford treatment with established, more senior practitioners. Although these clinics provide valuable treatment services, ethical concerns remain about the quality of medical care provided to those without insurance and other vulnerable groups [10]. These concerns include the possibility that SRFCs benefit the trainees, who gain much-valued experience, at the expense of disadvantaged patient populations who have no or limited alternative options; because student-trainees are not as experienced and skilled as more advanced practitioners, there is potential risk that they provide lower-quality, perhaps even sub-standard, care to those they serve [11].

Considering these important ethical concerns, the possibility of SRFCs providing sub-standard care must be evaluated empirically. Especially because such clinics most often serve highly vulnerable individuals, those working in SRFCs should evaluate the quality of the services they provide against accepted guidelines using empirically derived metrics. Data from two California clinics suggest that SRFCs can effectively identify depressive disorders and that those who received treatment had improvements in symptom severity from baseline [12]. A study from the Yale-affiliated HAVEN clinic found that students could be trained to provide psychoeducation and lay counseling to depressed patients that led to symptom improvement [13]. Our clinic has reported preliminary evidence that an SRFC could provide clinical services for depressed patients that were as good as or superior to those rendered to patients enrolled in public health insurance programs [14]; however, a later report [15] documented lower rates of effective acute- and continuation-phase antidepressant treatment. While a follow-up study [16] indicated that on-site antidepressant dispensing may improve adherence rates, it is not known if this led to overall improvements in the number of patients meeting criteria for effective acute- and continuation-phase treatment long-term. Furthermore, it has yet to be determined whether SRFCs can provide quality care on the many other relevant clinical performance metrics beyond antidepressant treatment. A recent report [17] demonstrated that transitioning an SRFC to a telepsychiatry treatment-delivery platform during the onset of the COVID-19 pandemic was positively received by patients, but the investigators did not evaluate the clinic’s comparative performance. Finally, no studies to date have reported long-term clinical outcomes among patients treated in these clinics.

For these reasons, we sought to investigate the comparative performance of our SRFC in the provision of mental healthcare services and the longitudinal outcomes of patients treated in our clinic. With this aim in mind, we had the following goals: (1) Describe the socio-demographic and psychiatric morbidity data among our patient population; (2) evaluate our clinic’s performance on behavioral healthcare service measures compared to that of clinics serving insured patients; (3) assess the extent to which patient’s depressive and anxious symptoms changed over time and the factors associated with differential treatment outcomes; and, (4) ascertain patients’ feedback on the clinical care they received. We hypothesized that the E-MHC would perform at levels non-inferior to those observed among New York State PPOs, HMOs, and Medicaid; furthermore, we predicted that patients PHQ-9 and GAD-7 scores would decrease as a function of the number of treatment sessions in the clinic. With the results obtained, we aimed to generate more comprehensive information on the psychiatric conditions treated in SRFCs, the comparative quality of clinical services provided for these conditions, the clinical outcomes that resulted, and patients’ perceptions of their own treatments and outcomes.

## Methods

### Setting

The East Harlem Health Outreach Partnership (EHHOP) is a student-run and faculty-supervised clinic affiliated with the Icahn School of Medicine at Mount Sinai in New York, NY. Established in 2004, EHHOP provides free primary care to East Harlem adults (22 years and older) who are unable to obtain health insurance, most often because they have not met legal residency requirements. In 2018, 12% of East Harlem adults reported not having insurance [18], and a 2017 study estimated that there were at least 14,000 immigrants living in East Harlem who did not meet residency requirements [19]. Compared to most other NYC neighborhoods, East Harlem has higher rates of unemployment, violent crime, and premature death and a rate of psychiatric hospitalizations that is three times the NYC average [18]. Demographically, 50% of East Harlem residents identify as Hispanic and 30% as black [18].

### Approach to the evaluation and treatment of psychiatric disorders

The EHHOP Mental Health Clinic (E-MHC) is a co-habiting clinic that accepts patients who receive primary care at the main medical clinic of EHHOP. Interdisciplinary management is key to its success, as student clinicians in the primary care clinic and E-MHC co-manage patients with a high prevalence of complex medical disease and psychiatric illness.

At initial intake to EHHOP and at least once annually, patients are screened for depressive and anxiety disorders using the Patient Health Questionnaire-9 (PHQ-9) [20] and the Generalized Anxiety Disorder-7 Scale (GAD-7) [21], respectively. Patients with positive screening results on either measure or who otherwise express mental health concerns are referred to the E-MHC for further evaluation and treatment as necessary. Clinical services provided to E-MHC patients include psychiatric assessment, medication management, non-specific supportive counseling, and individual psychotherapy conducted by supervised medical student trainees. New patients are seen at least once monthly for medication management and more frequently if they are receiving psychotherapeutic interventions. After stabilization, a minority of patients are transitioned to bi-monthly or quarterly follow-up visits.

Fourth-year psychiatric residents, volunteer psychiatrists, and supervising clinical psychologists oversee the services provided by second to fourth year medical students or MD-PhD students who have completed the first year of medical school. Following all E-MHC patient appointments, the student trainees present their patient to a supervising psychiatrist or fourth-year resident in psychiatry. Initial diagnoses are based upon unstructured interviews by the student, who then finalizes the diagnostic formulation with the supervising psychiatrist or resident. Supervisors review the patient’s status, formulate a treatment plan with the student, and provide additional mentoring in outpatient psychiatry. After these discussions, both the student and the supervisor meet with the patient to answer questions, review the assessment and treatment plan, and ensure that there are no safety concerns.

Patients expressing suicidality at any time are given a more thorough risk assessment by the supervisor; if needed, patients are taken to a nearby emergency room for continued monitoring and stabilization. As needed, on call psychiatry and medical faculty supervise trainees who triage phone calls; faculty provide necessary navigation of care and communication with emergency room and inpatient teams.

Psychotropic medications are prescribed by the supervising psychiatrist, and patients receive their medications with no out-of-pocket costs either at a Mount Sinai Hospital pharmacy or on-site immediately after their appointments [16]. Of note, there is a limited formulary of medications stratified by cost on a web-based application that providers consult when prescribing medications. In between the Saturdays on which the E-MHC is open, first and second year medical and graduate students manage the clinic’s schedule and coordinate follow-up visits and appointment reminders for all patients.

### Determination of demographic and clinical characteristics of the patient population

Age, race/ethnicity, and sex (male or female) were identified by review of patients’ electronic medical records from January 1st, 2009 to March 1st, 2020. Psychiatric diagnoses were collected from the patient’s charts and confirmed in provider notes; patients could have multiple diagnoses if they were concomitantly diagnosed or if different diagnoses were listed throughout the course of treatment. Psychiatric diagnoses collected included major depressive disorder, persistent depressive disorder, seasonal affective disorder, depression not otherwise specified (NOS), generalized anxiety disorder, panic disorder, social anxiety disorder, somatic symptom disorder, anxiety not otherwise specific (NOS), adjustment disorder, post-traumatic stress disorder, borderline personality disorder, substance use disorders, and persistent complex bereavement disorder. We also collected information about current and past sexual assault and intimate partner violence (SA/IPV) based upon review of provider notes. There were no pre-defined hypotheses tested, and only summary statistics are presented.

### Evaluation of mental healthcare service performance

We evaluated the quality of mental healthcare services at the E-MHC using the Healthcare Effectiveness Data and Information Set (HEDIS) performance metrics established by the National Committee on Quality Assurance. Many previous studies of clinical care performance utilize the HEDIS metrics, as they are empirically derived and objectively defined measures with specific criteria designed to operationalize each aspect of healthcare performance [22]. The New York State (NYS) Department of Health (DOH) mandates reporting of HEDIS metric data from all major managed care plans in the state and makes the data publicly available through the NYS DOH website and various published reports [23–25]. Managed care plans include insurance plans provided through Medicaid, Preferred Partner Organizations (PPOs), and Health Maintenance Organizations (HMOs). We refer the reader to the NYS DOH published reports for more information on the characteristics of each of these plan types and the specific plans included [24, 25]. We selected HEDIS behavioral healthcare metrics based upon those that were relevant to the clinical services provided by the E-MHC in the year 2019 and that could be calculated using the metric’s definition and the availability of patient data. We were able to compare our performance on metrics for “optimal provider contacts for treatment of depressive disorders,” “receipt of effective acute- and continuation-phase antidepressant treatment,” multiple metrics relating to smoking cessation intervention, and “follow-up care after ED visits for alcohol and other drug dependencies (AOD).” The definitions and criteria for having met each metric are as follows:

#### Optimal provider contacts for treatment of depressive disorders

Defined as the patient attending three or more follow-up visits within the first 3 months after an initial diagnosis of a depressive disorder; at least one of these visits must be with the provider overseeing medication-management [14]. We defined the date of initial diagnosis of a depressive disorder as the date listed in the electronic medical record visit note corresponding to the intake assessment visit. We confirmed attendance to follow-up visits by cross-referencing all patient encounters listed with the corresponding visits notes.

#### Receipt of effective acute-phase and continuation-phase antidepressant treatment

These two metrics were defined according to the HEDIS criteria outlined under “Antidepressant Medication Management (AMM),” which applies to adults ages 18 years or older with a diagnosed depressive disorder who were newly treated with an antidepressant medication [22]. “Effective Acute Phase Treatment” is defined as those who actively took a prescribed antidepressant medication for at least 12 weeks, and “Effective Continuation Phase Treatment” is defined as those who actively took a prescribed antidepressant medication for at least 6 months [22]. We determined whether patients were actively taking their antidepressant medications by reviewing all visit notes within the specified time period following initiation of the medication; patients were considered to have met each of the metric criteria if the visit notes explicitly stated that the patient self-reported full medication adherence on greater than 80% of visits within the corresponding time periods.

#### Smoking cessation

We evaluated three HEDIS metrics related to smoking cessation. “Advising Smokers and Tobacco Users to Quit” is defined as whether patients who were current smokers or tobacco users received any cessation advice during the past year. We first determined which patients were current smokers or tobacco users through chart review and then read through each visit note from the prior year to see if there was any indication that the provider gave cessation advice. “Discussing Cessation Medications” refers to current smokers or tobacco users to whom the provider expressed a recommendation to consider cessation medications (e.g., varenicline, bupropion, etc.) in the past year, which we assessed via chart review. Finally, “Discussing Cessation Strategies” refers to current smokers or tobacco users who discussed or were provided with information on cessation strategies or behavioral methods during the past year. We assessed whether patients had received such information via chart review and used a liberal definition of “cessation strategies or behavioral methods” to include informal advice during a visit on some of the different techniques or approaches that can be used to quit smoking, the provider indicating that they gave the patient print-outs with information on cessation approaches, and any more formal discussion of cessation strategies including provision of behavioral techniques and/or any psychotherapeutic interventions [22].

#### Follow-up care after ED visits for alcohol or other drug dependencies

This is defined as patients with an established substance use disorder to alcohol and/or other substances who had a visit to the emergency department (ED) related to their substance use and who attended a follow-up visit within a specified period of time after discharge from the ED or hospital. There are two rates reported, one for attendance to a follow-up visit within 30 days of ED visit and one for attendance to a follow-up visit within 7 days of an ED visit. We determined which patients had a diagnosed substance use disorder and then ascertained both ED visits and attendance to follow-up visits via chart review [22].

We hypothesized that the E-MHC would perform at levels non-inferior to those of NYS clinics grouped by insurance type. To conduct hypothesis-testing, we first retrieved data collected and published by the NYS DOH [23–25]. For the effective antidepressant medication management acute- and continuation-phase metrics, we also included our previously published data [15] in the comparisons to determine if the E-MHC’s performance improved over time. Due to the small sample size in our study and the unbalanced group sizes, Fisher exact tests were used to quantify the likelihood of patients having met the specific metric criteria between the E-MHC in 2019 and those from each of the comparator groups. Results are reported as odds ratios with 95% confidence intervals (CIs) and were considered significant if *p* < 0.05; if results were non-significant, they were operationalized to mean that the E-MHC performance was non-inferior to that of the comparator group.

### Assessment of factors associated with clinical outcomes

We longitudinally assessed depressive and anxious symptoms using the PHQ-9 and GAD-7, respectively, which were available from patients’ charts as part of routine care and symptom-monitoring. We first defined baseline symptom scores as either the score upon referral to the E-MHC or the score(s) reported at the initial assessment visit. Patients who had neither and had no scores reported within the first month of treatment were excluded from the analyses. End-point scores were defined as those achieved at the last recorded visit for the patient within the study period. We only included those who had at least mild PHQ-9/GAD-7 symptoms at baseline, defined as a score of 5 or greater on both scales [20, 21]. For all statistical tests, major depressive disorder, depression NOS, persistent depressive disorder, seasonal affective disorder, and persistent complex bereavement disorder were combined into a single diagnostic category of “depression.” Similarly, generalized anxiety disorder, panic disorder, social anxiety disorder, and somatic symptom disorder were combined into a composite diagnostic category of “anxiety.” Below, we describe the hypothesis-testing procedures conducted:

1. We first sought to evaluate the extent to which patients’ depressive and anxious symptoms changed over the course of their treatment in the E-MHC. We hypothesized that end-point scores on both PHQ-9 and GAD-7 would be significantly lower than baseline scores. We used within-subjects paired t-tests for all patients included and within groups of patients categorized by psychiatric condition treated.
2. We then endeavored to model patients’ depressive and anxious symptom severity overtime throughout the course of treatment and ascertain factors accounting for differences in symptom severity. We tested the hypothesis that the number of treatment sessions received by patients in the E-MHC would be positively associated with the magnitude of symptom improvement. To do so, we used each patient’s repeated, longitudinal PHQ-9 and GAD-7 scores throughout treatment and generated linear mixed effects models to determine whether treatment session number (independent variable) was associated with symptom severity score (raw score on the PHQ-9 or GAD-7) as the dependent variable. Session number was treated as the fixed effect, and patient ID was treated as the random effect. Subsequently, we sought to determine whether the inclusion of additional demographic or clinical variables improved the extent to which session number predicted symptom score; variables of interest included age, sex, baseline PHQ-9 score, baseline GAD-7 score, whether the patient had more than one psychiatric diagnosis, the number of psychiatric diagnoses at baseline, and whether the patient was listed as having (Y/N) depression, anxiety, adjustment disorder, alcohol use disorder, post-traumatic stress disorder, and/or intimate partner violence. Candidate models were evaluated using all-subsets regression with the *leaps* package in R. Bayesian Information Criterion (BIC) scores, a measure of information explained by each model that penalizes for overfitting [26], were used for variable-selection.
3. Finally, we sought to determine what factors, if any, were associated with the probability that patients achieved a clinically significant improvement in symptoms by the end of treatment. To do so, we employed the current consensus definition of a “clinically significant improvement” (CSI) as a reduction in symptom severity of 50% or more [26, 27]. We hypothesized that higher treatment session number would be positively associated with the likelihood of attaining CSI. Subsequently, we evaluated multi-factorial models that included various combinations of the demographic and clinical variables considered in the evaluation of longitudinal symptom severity models mentioned above. To test these hypotheses, we generated logistic regression models with the dependent variable as whether the patient achieved a CSI in the PHQ-9/GAD-7 scores (Y/N). Candidate models were selected using all subsets regression with the *bestglm()* function of the *bestglm* package [28]. The model with the best BIC score was selected. Finally, we graphically illustrated model predictions using the *predict()* function in R.

### Evaluation of patients’ feedback

We created a custom *Patient Feedback Survey* based upon frequently asked questions in the research literature on scales measuring patient satisfaction with mental healthcare services [29]. All questions were asked on a 5-point scale with possible answers of “Strongly Disagree,” “Disagree,” “Neutral,” “Agree,” and “Strongly Agree.” All feedback surveys were collected at the end of patient visits, and some patients completed the survey more than once over the course of their overall treatment in the E-MHC. A copy of the *Patient Feedback Survey* is provided in Supplementary File 1. We did not have any pre-defined hypotheses and provide only descriptive statistics on the results for each survey question.

## Results

### Demographic and clinical characteristics of the patient population

Data were available from 69 unique patients, including 47 females and 22 males. The mean patient age was 46.8 (SD: 11.8) years old, and female patients tended to be older than male patients by about 8 years (mean female age: 49.3; mean male age: 41.4; Welch’s two-sample t-test: *t* = 3.15, df = 58.6, *p* = 0.0026). Two (2.9%) patients were Afro-Caribbean, and the remaining (97.1%) were Hispanic (Table 1). All patients had a depressive, anxiety, trauma-based, substance use, and/or adjustment disorder. In order of decreasing frequency, the psychiatric disorders diagnosed among our patients were: depressive disorder (62.3%, *n* = 43), anxiety disorder (24.6%, *n* = 17), post-traumatic stress disorder (PTSD) (24.6%, *n* = 17), alcohol use disorder (20.3%, *n* = 14), adjustment disorder (14.5%, *n* = 10), and borderline personality disorder (2.90%, *n* = 2). The full breakdown of all psychiatric disorders is shown in Table 1. Consistent with extant data on the frequent comorbidity among psychiatric disorders [30], about half (49.3%, *n* = 34) of patients had multiple psychiatric conditions: 27 (39.1%) had two conditions, 5 (7.3%) had three, and 2 (2.9%) had four or more diagnoses listed in their charts. Finally, 8 (11.6%) patients had a history of intimate partner violence (IPV).

**Table 1:** Demographic and Clinical Data

	Male, n (% total)	Female, n (% total)	***Total***
*Total*	22 (31.9)	47 (68.1)	69 (100.0)
*Age*	*n (% total by gender)*		*n (% total)*
20-29	2 (9.1)	2 (4.2)	4 (5.8)
30-39	6 (27.3)	6 (12.8)	12 (17.4)
40-49	11 (50.0)	19 (40.4)	30 (43.5)
50-59	3 (13.6)	10 (21.3)	13 (18.8)
60-69	0 (0.0)	7 (14.9)	7 (10.1)
70-79	0 (0.0)	3 (6.4)	3 (4.3)
*Race/Ethnicity*			
Hispanic	21 (95.5)	46 (97.9)	67 (97.1)
Afro-Carribean	1 (4.5)	1 (2.1)	2 (2.9)
White/Caucasian	0 (0.0)	0 (0.0)	0 (0.0)
Native American	0 (0.0)	0 (0.0)	0 (0.0)
Native Hawaiian/Other Pacific Islander	0 (0.0)	0 (0.0)	0 (0.0)
Asian	0 (0.0)	0 (0.0)	0 (0.0)
*Psychiatric Diagnoses*			
Depressive Disorder	9 (40.9)	36 (76.6)	45 (65.2)
Major Depressive Disorder	3 (13.6)	17 (36.2)	20 (29.0)
Persistent Depressive Disorder	0 (0.0)	1 (2.1)	1 (1.4)
Bereavement/Persistent Grief	0 (0.0)	3 (6.4)	3 (4.3)
Depression Not Otherwise Specified	4 (18.2)	15 (31.9)	20 (29.0)
Depression Due to a Medical Condition	1 (4.5)	0 (0.0)	1 (1.4)
Depression Due to a Substance	1 (4.5)	0 (0.0)	1 (1.4)
Anxiety Disorder	7 (31.8)	14 (29.8)	21 (30.4)
Generalized Anxiety Disorder	4 (18.2)	7 (14.9)	11 (15.9)
Panic Disorder	2 (9.1)	3 (6.4)	5 (7.2)
Somatic Symptom Disorder	0 (0.0)	2 (4.2)	2 (2.9)
Social Anxiety Disorder	1 (4.5)	0 (0.0)	1 (1.4)
Anxiety Not Otherwise Specified	0 (0.0)	2 (4.2)	2 (2.9)
Adjustment Disorder	2 (9.1)	8 (17.0)	10 (14.5)
Alcohol Use Disorder	14 (63.6)	0 (0.0)	14 (20.3)
Borderline Personality Disorder	0 (0.0)	2 (4.2)	2 (2.9)
Post-Traumatic Stress Disorder	4 (18.2)	13 (27.7)	17 (24.6)
Multiple Psychiatric Disorders	11 (50.0)	23 (48.9)	34 (49.3)
Intimate Partner Violence & Sexual Assault	0 (0.0)	8 (17.0)	8 (11.6)

### Mental healthcare service performance in the E-MHC

#### Optimal provider contacts for treatment of depressive disorders

**Fig. 1:**
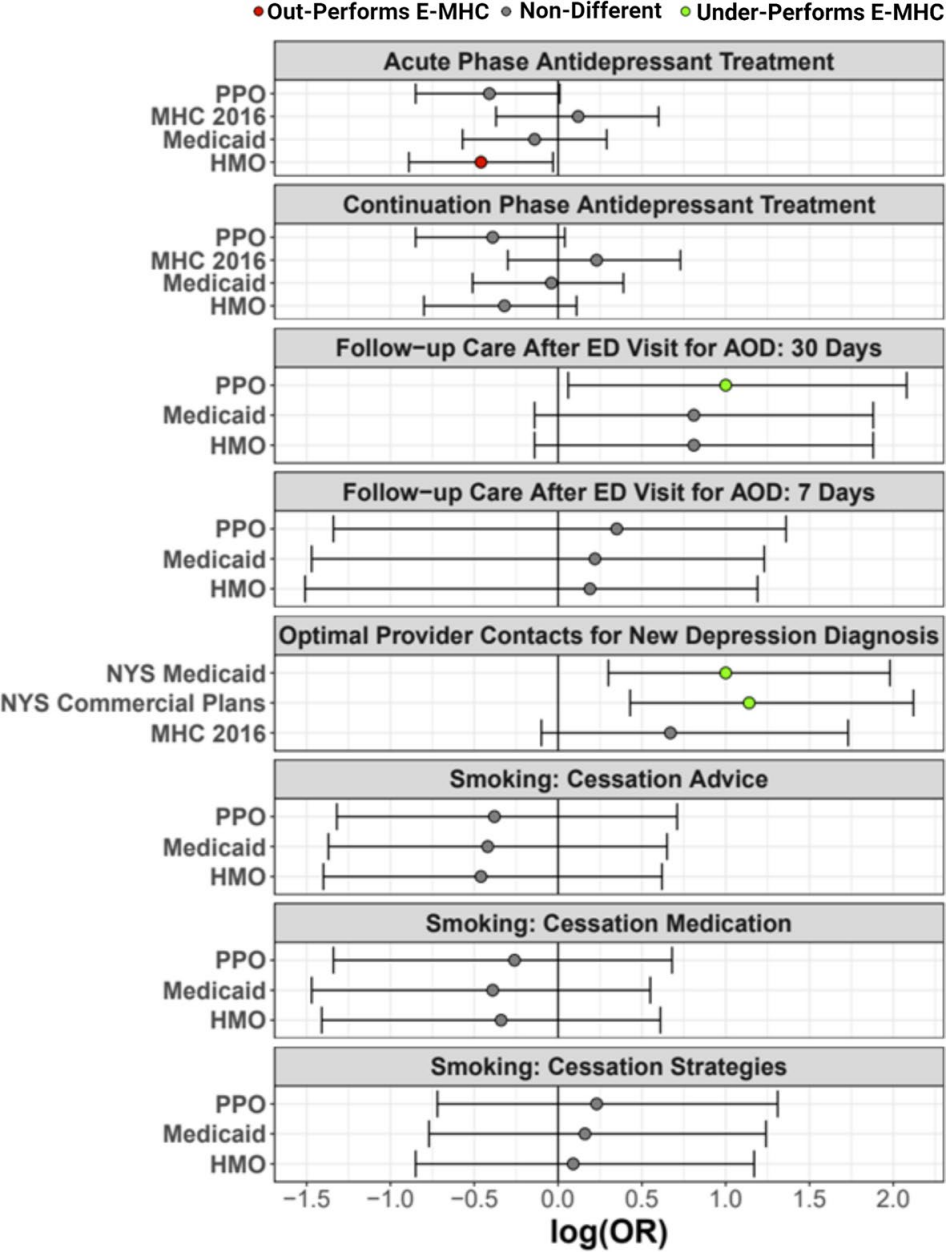
Performance quality in the provision of behavioral healthcare services by the E-MHC compared to various public and private New York State (NYS) insurance programs. Performance is operationalized as the odds ratio of a patient meeting criteria for a given performance metric compared to those in the insurance group indicated on the y-axis. Gray point-estimates of the log-transformed ORs indicate no evidence of a difference, while red indicates that the E-MHC underperforms the group shown and green indicates that the E-MHC out-performs the group shown

There were 10 new E-MHC patients with a depressive disorder diagnosis in 2019, 8 (80%) of whom met the criteria for optimal provider contacts. Comparisons to the proportion of patients receiving optimal provider contacts in overall NYS commercial plans and NYS Medicaid indicated that the E-MHC outperformed on this measure in both comparisons (Fig. 1). E-MHC patients had 13.7-times higher odds (95% CI: 2.7-132.9; *p* = 0.0002) of receiving optimal provider contacts than those in NYS commercial plans and 9.9-times higher odds (95% CI: 2.0-96.1; *p* = 0.0012) than those with NYS Medicaid. We also found suggestive evidence that depressed E-MHC patients in 2019 were more likely to receive optimal provider contacts than they were previously in our clinic [14] (80% versus 45%; OR = 4.7; 95% CI: 0.8 – 53.3; *p* = 0.074), indicating that the clinic may have improved over time on this important outcome.

#### Receipt of effective acute-phase and continuation-phase antidepressant treatment

We found that the proportion of depressed patients satisfying criteria for having received effective acute-phase antidepressant treatment was lower in the E-MHC than in patients served by NYS HMO programs (45% versus 70%; OR = 0.35, 95% CI: 0.13 – 0.93; *p* = 0.025); suggestive evidence supported a similar pattern compared to NYS PPO programs (45% versus 68%; OR = 0.39, 05% CI: 0.14 – 1.02; *p* = 0.051) (Fig. 1). We did not detect differences in the likelihood that E-MHC patients received effective acute-phase antidepressant treatment compared to those served by NYS Medicaid. In the case of effective *continuation*-phase antidepressant treatment, the E-MHC did not differ from NYS HMOs, PPOs, or Medicaid (Fig. 1).

#### Smoking cessation

Few patients in the E-MHC were documented as current smokers (*n* = 5). Across the three HEDIS performance metrics of (1) “advising smokers and tobacco users to quit,” (2) “discussing cessation medications”, and (3) “discussing cessation strategies”, we did not find evidence that the E-MHC performed differently than NYS HMO, PPO, and Medicaid programs (Fig. 1).

#### Follow-up care after ED visits for alcohol or other drug (AOD) dependencies

Rates of follow-up care within 7 and 30 days after an ER visit for AOD abuse or dependency for E-MHC patients (*n* = 5) were 20 and 60%, respectively, while the corresponding rates among Medicaid patients were 13 and 19%, 10 and 13% among PPOs, and 14 and 19% among HMOs. We did not detect pairwise differences in the likelihood of receiving 7-day follow-up between the E-MHC and Medicaid, PPOs, or HMOs (Fig. 1). The likelihood of appropriate follow-up within 30 days of ER visit, however, was higher in the E-MHC compared to rates in all three comparison groups: Medicaid (OR = 6.39; 95% CI: 0.73 – 76.45; *p* = 0.051); PPOs (OR = 10.04; 95% CI: 1.15 – 120.29; *p* = 0.018); HMOs (OR = 6.39, 95% CI: 0.73 – 76.47; *p* = 0.051) (Fig. 1).

### Factors associated with clinical outcomes

**Fig. 2:**
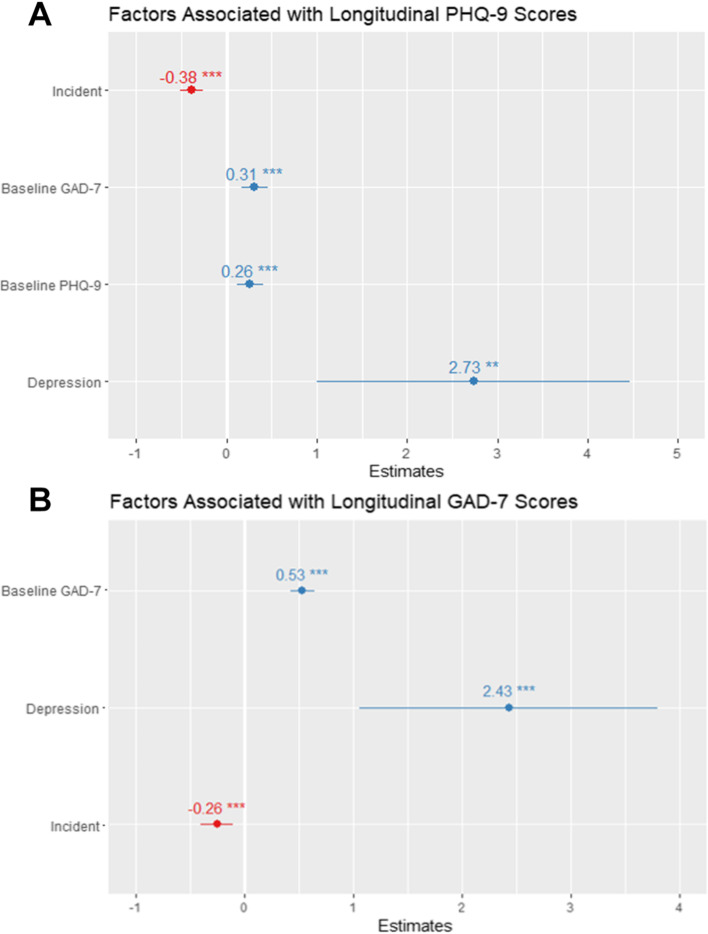
Modeling longitudinal change in depressive and anxious symptom severity over time. **A** Coefficients for the fixed effects in the LME model accounting for the change in PHQ-9 over time. Both baseline PHQ-9 and GAD-7 predict higher depressive symptom severity, as does a depressive disorder diagnosis. In contrast, incident session number predicts lower symptom severity. **B** Coefficients for the fixed effects in the LME model accounting for the change in GAD-7 over time. Baseline GAD-7 and a depressive disorder diagnosis predict worse anxious symptom severity while incident session predict lower anxious symptom severity

Finally, we investigated the factors associated with long-term outcomes among patients treated in the E-MHC. Across diagnoses, there were significant pre-post improvements in the magnitude of depressive and anxious symptoms (see Supplementary Fig. 1). We found that the session number predicted lower symptom severity over the course of treatment on both PHQ-9 and GAD-7, thus providing robust evidence of a specific treatment effect of sessions in the E-MHC. Furthermore, baseline PHQ-9, baseline GAD-7, and a diagnosis of a depressive disorder predicted higher depressive symptom severity throughout the treatment period (Fig. 2). Longitudinal modeling of GAD-7 scores revealed a similar pattern, with baseline GAD-7 (but not baseline PHQ-9) and diagnosis of a depressive disorder predicting higher anxious symptom severity (Fig. 2).

**Fig. 3:**
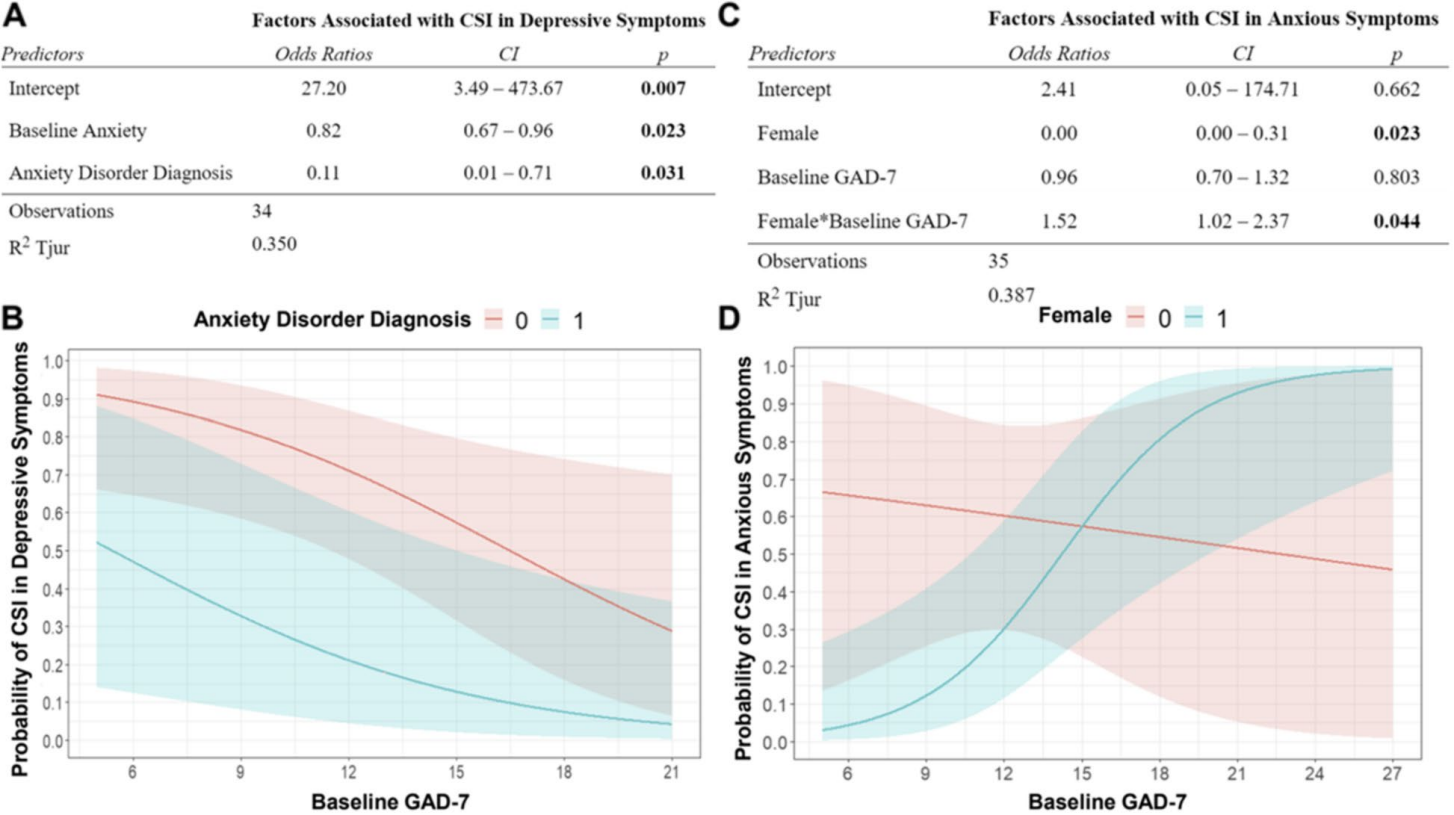
Logistic Regression modeling of likelihood of clinically significant improvement in depressive and anxiety symptom scores. **A** Baseline GAD-7 score and diagnosis of an anxiety disorder predict lower odds of a clinically significant improvement in depression symptom severity. **B** Graphical illustration of the predicted impact of baseline GAD-7 and the presence versus absence of an anxiety disorder diagnosis based upon simulation data. **C** Interaction of female sex with baseline GAD-7 score in the likelihood of clinically significant improvement in anxiety. **D** There is little relationship between baseline score and likelihood of improvement in anxious symptoms for men, but a clear sigmoidal curve for female patients showing a higher predicted likelihood of improvement with increasing baseline GAD-7 score

For models of those factors associated with likelihood of achieving a clinically significant improvement in depressive symptom severity, we found that it was baseline GAD-7, and not baseline PHQ-9, as well as diagnosis of an anxiety disorder that were associated with lower likelihood of a clinically significant improvement (Fig. 3). For clinically significant improvement in anxious symptoms, an initial model consisting of GAD-7 score at baseline had the best performance. However, upon inspecting the individual datapoints grouped by sex, we noticed that the effect of baseline GAD-7 score appeared to differ remarkably between male and female patients. A model that included an interaction term revealed that the effect of baseline anxious symptom severity varied by sex. Simulation results demonstrated little relationship between baseline GAD-7 score and probability of improvement in males, whereas a sigmoidal relationship was found between baseline GAD-7 and probability of improvement in females (Fig. 3).

### Patients’ feedback to E-MHC care

**Fig. 4:**
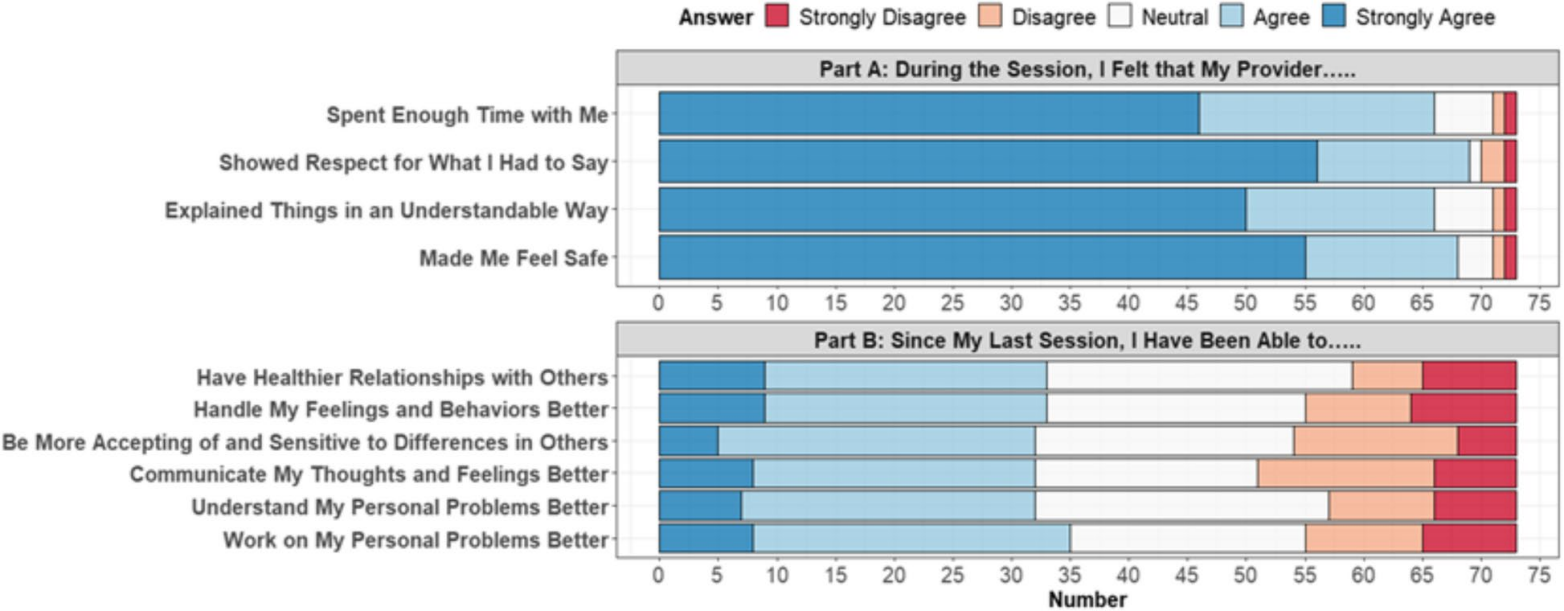
Results from *n* = 73 surveys show that patients receiving care at an outpatient psychiatric SRFC provide highly positive feedback. (Top) In Part **A** of the *Patient Feedback Survey*, subjects were asked to rate various aspects of the provision of care by their student providers. Results show that the vast majority of patients either agreed or strongly disagreed that their student provider spent enough time with them, showed respect for what they had to say, explained things in an understandable way, and made them feel safe. (Bottom) In Part **B** of the *Patient Feedback Survey*, subjects were asked to self-report on their perceived improvement in broad domains of mental health and psychosocial functioning compared to their prior session. About half reported improvements across the various domains

We finally set out to ascertain patients’ feedback on the care they received in our clinic and their self-reported perceptions of overall improvement in broad domains of psychosocial functioning. In sum, a total of 73 patient feedback surveys were collected. As shown in Fig. 4, patients provided highly positive feedback on the care they received from their student providers (Part A). Results from Part B of the survey demonstrated that patients also perceived that they improved in broad dimensions of their mental health and psychosocial functioning (Fig. 4). Overall, these results clearly show that the outpatient mental healthcare services rendered by student providers in the E-MHC are highly well-received by patients.

## Discussion

In this study, we sought to describe the design of an outpatient psychiatric SRFC integrated within a comprehensive primary care clinic and empirically determine the demographic and clinical characteristics of the population served, the performance quality of mental healthcare services provided, the clinical outcomes attained and factors associated thereof, and finally, patient feedback to the care received.

### Demographic and clinical characteristics of the patient population

We provide important data on the prevalence of common psychiatric disorders encountered in an outpatient setting serving uninsured immigrants in the East Harlem community. Our findings on psychiatric disorder prevalence rates and comorbidity are generally consistent with results from large U.S. studies [30]. As such, they provide evidence that broad patterns of disorder prevalence are not substantively different among uninsured immigrant populations and native U.S. samples and thus inform inferences about the conditions most likely to be encountered in this clinical population. Our finding that female patients were older than male patients may be driven by multiple factors. Further investigation is needed to determine whether this sex difference is due to differences in the age of initial presentation for treatment, receipt of consistent follow-up, and/or social factors affecting patient access to and ability to undergo psychiatric treatment.

### Mental healthcare service performance in the E-MHC

Upon evaluating the quality of services provided by the E-MHC in 2019, we found that patients in our clinic were more likely to receive the optimal number of provider contacts following a diagnosis of a depressive disorder than were patients insured by NYS commercial plans and Medicaid. Additionally, we found suggestive evidence that patients were more likely to have received optimal provider contacts in our clinic in 2019 compared to the data reported from the previous EHHOP study, which covered the years 2004-2009 [14]. This is likely because it was not until 2008 that a separate, outpatient mental health clinic was founded within EHHOP, along with the substantial expansion of the number and kinds of services we have provided since.

In terms of the proportions of patients receiving effective acute- and continuation-phase antidepressant treatment, we found evidence that E-MHC patients were less likely to have received effective acute-phase antidepressant medication treatment than were HMO patients; an identical finding almost reached statistical significance when comparing the E-MHC to PPOs. No differences were detected between the E-MHC and Medicaid. Of note, we found that the E-MHC was non-inferior to NYS Medicaid, PPOs, and HMOs on effective continuation-phase antidepressant treatment, whereas our previous study found the E-MHC to be inferior on this metric [15]. These findings indicate only somewhat of an improvement over our previously reported findings [15] that spanned 2009-2016. Comparisons between the present data and that from our previous report [15], however, did not yield sufficient evidence that rates of antidepressant adherence have improved. Even though we noted in a recent report [16] that on-site prescription dispensing improved adherence rates at the level of individual patient visits as well as overall percent adherence rates per patient, that study did not utilize the specific HEDIS metric criteria (84 days for acute-phase management and 180 days for continuation-phase management). With the present results in mind, it appears that larger sample sizes and/or additional interventions are needed to demonstrate improvement in our clinic over time.

This study is the first report of performance metrics of an outpatient psychiatry SRFC on clinical services related to tobacco-smoking and alcohol abuse. Although limited by small sample sizes, we did not find evidence that the E-MHC differed from NYC PPOs, HMOs, and Medicaid in the rates of advising smokers to quit, discussing cessation medications, and discussing cessation strategies. The E-MHC outperformed NYS PPO, HMO, and Medicaid groups on appropriate follow-up visits after an alcohol- or other drug-related ED visit within 30 but not 7 days. These limited and preliminary results warrant expanded study in our clinic and other SRFCs.

Broadly, the results obtained in our analyses of mental healthcare service performance support the hypothesis that properly trained and supervised students can provide quality clinical care that is generally non-inferior to that achieved in NYS managed care plans. This has important implications for healthcare policy, especially during a time in which the demand for mental health services is great and existing clinical infrastructures are challenged to meet it [2].

### Factors associated with clinical outcomes

The results from our performance analyses are complemented by assessment of factors explaining clinical outcomes in our clinic. Indeed, in the longitudinal course of both depressive and anxious symptom severity, we found that higher treatment session number in the E-MHC predicted lower scores, directly suggesting a treatment effect of our clinic. In terms of the likelihood of patients attaining a clinically significant improvement in depressive symptoms, baseline anxiety symptoms and diagnosis of an anxiety disorder both predicted lower odds. These findings may be due in part to the fact that co-morbid psychiatric diagnoses can decrease the likelihood of positive clinical responses in the treatment of depressive conditions [31]. However, our analysis of clinically significant improvement in anxiety symptoms found a strong effect of higher baseline anxiety symptoms increasing the likelihood of a response in females but not males. To our knowledge, sex-specific modification of the effect of baseline symptom severity on likelihood of response has not been previously reported. These results suggest the possibility of a sex-specific effect on the utility of baseline anxious symptom severity in predicting likelihood of overall treatment response.

### Patients’ feedback on mental healthcare services received in the E-MHC

Finally, results from our feedback surveys demonstrate strongly that patients viewed the clinical services they received in the E-MHC positively. These findings are important, as little data exist on the perceived quality of services received from psychiatric SRFCs. Our results support the notion that SRFCs can provide outpatient psychiatric care that is well-received by patients. Our group’s previous study [17] found that provision of SRFC mental health services via a telepsychiatry platform was also highly well received by patients, who felt that it helped them manage their symptoms of depression and anxiety during the initial months of the COVID-19 pandemic.

### Study limitations

The primary limitation of this study is its small sample size. While the number of patients included in this study is comparable to prior reports [12, 13, 15], larger and multi-site studies are needed to more robustly characterize the demographic and clinical psychiatric profiles of low-resource patients served at SRFCs; such studies are also needed to establish evidence-based best-practice guidelines for student training and the provision of behavioral health services in SRFCs. Furthermore, the structure of the E-MHC as part of an integrated system of clinics serving chronically ill patients had important effects on our sampling plan, and therefore, the generalizability of our findings. Here, the clinical population under study is not just particularly vulnerable due to uninsured immigrant status and the associated psycho-social-economic difficulties, but also because of the high rates of complex medical diseases in our patients. As such, the generalizability of the findings reported herein may be limited more to those with high medical comorbidities than to outpatient psychiatric patients overall. Despite these limitations, we believe this study is an important advancement in establishing empirical approaches to evaluating the clinical outcomes and treatment service quality in an SRFC serving vulnerable patients.

## Supplementary Information

**Additional file 1: Supplementary Fig. 1.** Composite changes from baseline in the severity of depressive symptoms (Supplementary Fig.1A) and anxious symptoms (Supplementary Fig.1B) among patients with at least 3 treatment sessions in the E-MHC. Results are grouped by all patients as well as across specific psychiatric diagnoses or problems. “Multi.Diags” refers to patients with more than one psychiatric diagnosis. “AUD” = alcohol use disorder. “AD” = adjustment disorder. “IPV” = intimate partner violence and/or sexual assault. “PTSD” = post-traumatic stress disorder. Individual, patient-level data points are shown with lines connect the baseline score (red dots) to the endpoint score (teal dots), overlaid on boxplots and violin plots showing the distributions and spread of the data. NS = “not significant.” * indicates *p* < 0.05; ** indicates *p* < 0.01; *** indicates *p* < 0.001, and **** indicates *p* < 0.00001. All tests conducted as paired t-tests.

**Additional file 2: Supplementary File.** *Patient Feedback Survey* in English

## Data Availability

All primary data and analysis scripts (in R) are available from the corresponding author upon request.

## Abbreviations

EHHOP: East Harlem Health Outreach Partnership
MHC: Mental Health Clinic
E-MHC: East Harlem Health Outreach Partnership Mental Health Clinic
NYC: New York City
NYS: New York State
HMO: Health Maintenance Organization
PPO: Preferred Provider Organization
SRFC: Student-Run Free Clinic
HEDIS: Health Effectiveness Data and Information Set
PHQ-9: Patient Health Questionnaire 9
GAD-7: Generalized Anxiety Disorder 7
IPV: Intimate Partner Violence
AUD: Alcohol Use Disorder
PTSD: Post-Traumatic Stress Disorder
ED: Emergency Department
BIC: Bayesian Information Criterion
