# Supplementary figures and images for: Design of and outcomes in a student-run free mental health clinic serving the uninsured in East Harlem
Source: BMC Psychiatry. 2022 Jul 26;22:501. doi: 10.1186/s12888-022-04112-w (PMC9321276; doi:10.1186/s12888-022-04112-w)

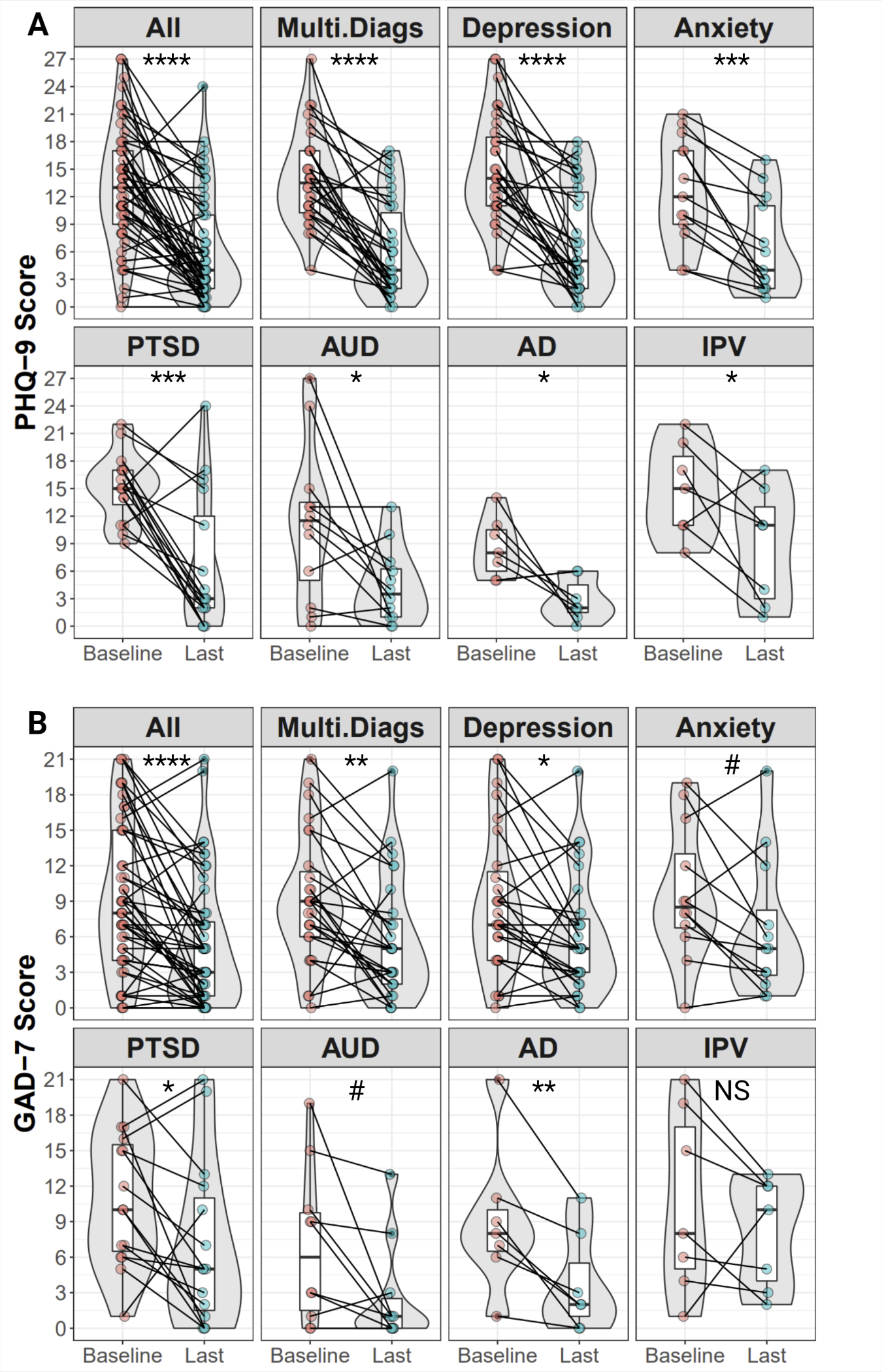

Supplement: Supplementary file 1 — Additional file 1: Supplementary Fig. 1. Composite changes from baseline in the severity of depressive symptoms (Supplementary Fig.1A) and anxious symptoms (Supplementary Fig.1B) among patients with at least 3 treatment sessions in the E-MHC. Results are grouped by all patients as well as across specific psychiatric diagnoses or problems. “Multi.Diags” refers to patients with more than one psychiatric diagnosis. “AUD” = alcohol use disorder. “AD” = adjustment disorder. “IPV” = intimate partner violence and/or sexual assault. “PTSD” = post-traumatic stress disorder. Individual, patient-level data points are shown with lines connect the baseline score (red dots) to the endpoint score (teal dots), overlaid on boxplots and violin plots showing the distributions and spread of the data. NS = “not significant.” * indicates p < 0.05; ** indicates p < 0.01; *** indicates p < 0.001, and **** indicates p < 0.00001. All tests conducted as paired t-tests. [file 12888_2022_4112_MOESM1_ESM.jpeg]
