# Supplementary material for: Design of and outcomes in a student-run free mental health clinic serving the uninsured in East Harlem
Source: BMC Psychiatry. 2022 Jul 26;22:501. doi: 10.1186/s12888-022-04112-w (PMC9321276; doi:10.1186/s12888-022-04112-w)
Supplement: Supplementary file 2 — Additional file 2: Supplementary File. Patient Feedback Survey in English [file 12888_2022_4112_MOESM2_ESM.pdf]

# MHC- general patient feedback

We would like feedback on your experience. This information is voluntary and will be kept confidential. We appreciate your honesty and ask that you do not put your name on the sheet so that your responses will remain anonymous. Your feedback will help us improve our services.

**1. MRN IDENTIFIER #:**

______________________________

**1. During your session, did you feel that the people you went to for counseling or treatment explain things in a way you could understand?**

- ○ Strongly Agree
- ○ Agree
- ○ Neutral
- ○ Disagree
- ○ Strongly Disagree

**2. During your session, did you feel that the people you went to for counseling or treatment show respect for what you had to say?**

- ○ Strongly Agree
- ○ Agree
- ○ Neutral
- ○ Disagree
- ○ Strongly Disagree

**3. During your session, did you feel that the people you went to for counseling or treatment spend enough time with you?**

- ○ Strongly Agree
- ○ Agree
- ○ Neutral
- ○ Disagree
- ○ Strongly Disagree

**4. During your session, did you feel safe when you were with the people you went to for counseling or treatment?**

- ○ Strongly Agree
- ○ Agree
- ○ Neutral
- ○ Disagree
- ○ Strongly Disagree

**5. During your session, were you prescribed any prescription medicines as part of your treatment?**

- ◯ Yes
- ◯ No

**6. Were you told what side effects of those medicines to watch for?**

- ◯ Yes
- ◯ No
- ◯ N/A

**7. Using any number from 0 to 10,where 0 is the worst counseling or treatment possible and 10 is the best counseling or treatment possible, what number would you use to rate this counseling or treatment session?**

- ◯ 0 (worst counseling or treatment possible)
- ◯ 1
- ◯ 2
- ◯ 3
- ◯ 4
- ◯ 5
- ◯ 6
- ◯ 7
- ◯ 8
- ◯ 9
- ◯ 10 (Best counseling or treatment possible)

8.Did any of the following make any difference in the counseling or treatment you need? Please circle one for each:

**Language**

- ◯ Yes
- ◯ No

**Race**

- ◯ Yes
- ◯ No

**Religion**

- ◯ Yes
- ◯ No

**Culture**

- ◯ Yes
- ◯ No

**Ethnic Background**

- ◯ Yes
- ◯ No

**9. In general, how would you rate your overall mental health now?**

- ◯ Excellent
- ◯ Very Good
- ◯ Good
- ◯ Fair
- ◯ Poor

**10. Compared to before this session, how would you rate your ability to deal with daily problems now?**

- ◯ Much better
- ◯ A little better
- ◯ About the same
- ◯ A little worse
- ◯ Much worse

**11. Compared to before this session, how would you rate your ability to deal with social situations now?**

- ◯ Much better
- ◯ A little better
- ◯ About the same
- ◯ A little worse
- ◯ Much worse

**12. Compared to before this session, how would you rate your ability to accomplish the things you want to do now?**

- ◯ Much better
- ◯ A little better
- ◯ About the same
- ◯ A little worse
- ◯ Much worse

**13. Compared to before this session, how would you rate your problems or symptoms now?**

- ◯ Much better
- ◯ A little better
- ◯ About the same
- ◯ A little worse
- ◯ Much worse

**14. Further comments on any of the above scales or about your counseling experience at EHHOP MHC**

______________________________________________________________________
